# Multiparametric computer-aided differential diagnosis of Alzheimer’s disease and frontotemporal dementia using structural and advanced MRI

**DOI:** 10.1007/s00330-016-4691-x

**Published:** 2016-12-16

**Authors:** Esther E. Bron, Marion Smits, Janne M. Papma, Rebecca M. E. Steketee, Rozanna Meijboom, Marius de Groot, John C. van Swieten, Wiro J. Niessen, Stefan Klein

**Affiliations:** 1 Biomedical Imaging Group Rotterdam, Departments of Medical Informatics and Radiology & Nuclear Medicine, Erasmus MC, Office Na2502, P.O. Box 2040, 3000 CA Rotterdam, The Netherlands; 2 Department of Radiology & Nuclear Medicine, Erasmus MC, Rotterdam, The Netherlands; 3 Department of Neurology, Erasmus MC, Rotterdam, The Netherlands; 4 Department of Epidemiology, Erasmus MC, Rotterdam, The Netherlands; 5 Imaging Physics, Applied Sciences, Delft University of Technology, Delft, The Netherlands

**Keywords:** Classification, Dementia, Differential diagnosis, Perfusion, Diffusion tensor imaging

## Abstract

**Objectives:**

To investigate the added diagnostic value of arterial spin labelling (ASL) and diffusion tensor imaging (DTI) to structural MRI for computer-aided classification of Alzheimer's disease (AD), frontotemporal dementia (FTD), and controls.

**Methods:**

This retrospective study used MRI data from 24 early-onset AD and 33 early-onset FTD patients and 34 controls (CN). Classification was based on voxel-wise feature maps derived from structural MRI, ASL, and DTI. Support vector machines (SVMs) were trained to classify AD versus CN (AD-CN), FTD-CN, AD-FTD, and AD-FTD-CN (multi-class). Classification performance was assessed by the area under the receiver-operating-characteristic curve (AUC) and accuracy. Using SVM significance maps, we analysed contributions of brain regions.

**Results:**

Combining ASL and DTI with structural MRI resulted in higher classification performance for differential diagnosis of AD and FTD (AUC = 84%; *p* = 0.05) than using structural MRI by itself (AUC = 72%). The performance of ASL and DTI themselves did not improve over structural MRI. The classifications were driven by different brain regions for ASL and DTI than for structural MRI, suggesting complementary information.

**Conclusions:**

ASL and DTI are promising additions to structural MRI for classification of early-onset AD, early-onset FTD, and controls, and may improve the computer-aided differential diagnosis on a single-subject level.

***Key points*:**

• *Multiparametric MRI is promising for computer-aided diagnosis of early-onset AD and FTD.*

*• Diagnosis is driven by different brain regions when using different MRI methods.*

*• Combining structural MRI, ASL, and DTI may improve differential diagnosis of dementia.*

**Electronic supplementary material:**

The online version of this article (doi:10.1007/s00330-016-4691-x) contains supplementary material, which is available to authorized users.

## Introduction

Alzheimer's disease (AD) and frontotemporal dementia (FTD) are major diseases underlying dementia, especially in younger patients (age < 65 years) [1]. Establishing an accurate diagnosis in the early stage of the disease can be difficult. Although clinical symptomatology differs between the diseases, symptoms in the early stage may be unclear and can overlap [2, 3]. The current clinical criteria, which entail qualitative inspection of neuroimaging, fail to accurately differentiate AD from FTD [4]. However, early and accurate differential diagnosis of AD and FTD is very important, mainly because it gives patients access to supportive therapies [5, 6]. In addition, early diagnosis supports new research into understanding the disease process and developing new treatments [5, 6].

In this difficult case of differential diagnosis between AD and FTD, methods for computer-aided diagnosis may be beneficial. These methods make use of multivariate data analysis techniques that train a model (classifier) based on neuroimaging or related data, resulting in an objective diagnosis. In addition, computer-aided diagnosis can be more accurate than using only clinical criteria [7], as it potentially makes use of subtle group differences. Using structural T1-weighted (T1w) MRI to find characteristic patterns of brain atrophy, computer-aided diagnosis methods yielded accuracy of up to 84% for differentiation of AD and FTD [8–10].

Besides using structural MRI, evidence of neurodegeneration can be measured with advanced MRI techniques such as arterial spin labelling (ASL) and diffusion tensor imaging (DTI). ASL can non-invasively measure brain perfusion in terms of cerebral blood flow (CBF) [11, 12]. Recent studies have shown differences in perfusion patterns for FTD and AD indicating that this technique is promising for differential diagnosis [13–16]. In addition, some classification studies showed an added value of ASL over atrophy measurements for AD diagnosis in individual patients, although others did not [13, 17–19]. Using DTI, the fractional anisotropy (FA) can be quantified, which is related to the degradation of white matter (WM) bundles. WM degradation has been shown to be more prominent in FTD than in AD, especially in frontal brain regions [14, 20, 21]. In classification studies, DTI generally shows a slight added value to atrophy measurements [22–28].

As ASL and DTI measure aspects of the neurodegenerative process that are different from brain volume changes, we hypothesise that these techniques have an added diagnostic value over structural MRI. Although ASL and DTI have been shown to be potential markers for differential diagnosis of AD and FTD, their combined added value for computer-aided differential diagnosis has not yet been evaluated. This study aims to investigate the added diagnostic value of ASL and DTI to structural MRI for classification of AD, FTD, and controls.

## Materials and methods

### Participants

We retrospectively included 24 AD patients, 33 FTD patients, and 34 cognitively normal (CN) controls. Patients who visited the memory clinic of our institution between February 2011 and June 2015 were considered for inclusion. Patients underwent neurological and neuropsychological examination as part of their diagnostic work-up. Patients with a Mini-Mental State Examination (MMSE) score ≥ 20 were included if they had undergone MR imaging with a standardised protocol including structural T1w MRI, ASL, and DTI. Patients with psychiatric or neurological disorders other than dementia were excluded. The reference standard was a diagnosis of AD or FTD established by consensus of a multidisciplinary team according to the clinical criteria [2, 3, 29]. Controls were recruited from patient peers and through advertisement, and had no memory complaints, history of neurological or psychiatric disease, or contra-indications for MRI.

This study was approved by the local medical ethics committee. Eighty-seven participants signed informed consent; consent from the remaining four patients was waived because of the retrospective nature of the study.

### Image acquisition and processing

MR imaging was performed at 3 T with 8-channel head coils on two identical scanners (Discovery MR750; GE Healthcare, Milwaukee, WI, USA). The protocol included T1w, ASL, and DTI. High-resolution isotropic T1w images were acquired with 3D inversion recovery fast spoiled gradient-recalled echo. According to the recommendations for ASL [12], we acquired 3D pseudo-continuous ASL perfusion-weighted images and a separate proton-density image for scaling. DTI used 2D single-shot echo planar imaging in 25 non-collinear directions [30]. Detailed parameters are listed in Table 1.

**Table 1:** MRI acquisition parameters

	T1w	ASL	DTI
Sequence	3D IR FSPGR	3D pCASL	2D single-shot EPI
Scan parameters (TI/TR/TE)	450 ms / 7.9 ms / 3.1 ms	1525 ms^a^ / 4632 ms / 10.5 ms	N.A. / 7925 ms / 82 ms
Resolution	1 mm isotropic	3.3 mm isotropic	1.9 × 1.9 in-plane
Acquisition matrix	240 × 240 × 176	512 sampling points on 8 spirals	128 × 128
Reconstructed voxel size	0.9 × 0.9 × 1.0 mm (sagittal)	1.9 × 1.9 × 4.0 mm (axial)	0.9 × 0.9× 2.5 mm or 0.9 × 0.9 × 2.9 mm (axial)
*ASL-specific*			
Labelling duration	-	1450 ms	-
Number of excitations	-	3	-
Background suppression	-	Yes	-
Readout	-	Interleaved fast spin echo	-
*DTI-specific*			
Non-collinear directions	-	-	25
Maximum b-value	-	-	1000 s/mm2
No. b_0_ volumes (b-value = 0 s/mm^2^)	-	-	3
Acquisition time	4:41 min	4:29 min	4:50 min

^a^For ASL, TI equals the post-labelling delay

*ASL* arterial spin labelling, *DTI* diffusion tensor imaging, *EPI* echo-planar imaging, *FSPGR* fast spoiled gradient-recalled echo, *IR* inversion recovery, *pCASL* pseudo-continuous ASL, *T1w* structural T1-weighted MRI, *TE* echo time, *TI* inversion time, *TR* repetition time

For image processing, the *Iris pipeline* [19] was applied to obtain voxel-based measures of structural MRI, ASL, and DTI (see Appendix A for a detailed description). From structural MRI, we derived tissue segmentations—WM, grey matter (GM), cerebrospinal fluid—and a brain mask. In a group template space, we derived features based on voxel-based morphometry (VBM) within a mask of the 1) GM (*VBM-GM*), 2) WM (*VBM-WM*) and 3) supratentorial brain (*VBM-Brain*). For ASL, CBF was quantified using a single-compartment model and partial volume correction. The CBF voxel values of the GM in the template space were used as features for classification. For DTI, tensor fits were performed to derive FA maps. The FA voxel values in WM in the template space were used as features for classification.

### Quality control

The following images were visually inspected (E.E.B., 5 years of experience): GM segmentation, WM segmentation, brain mask, template space registration, ASL registered to structural MRI, CBF map, DTI registered to structural MRI, and FA map. Any errors in the image processing were corrected until visual inspection revealed no more unacceptable results.

### Analysis and statistics

Classifications of AD versus CN (AD-CN), FTD-CN, and AD-FTD were performed with linear support-vector-machine (SVM) classifiers [31]. The SVM C-parameter was optimised in cross-validation on the training set. Classifiers were trained on *VBM-GM*, *VBM-WM*, *VBM-Brain*, *CBF*, and *FA* features separately. For combination of multiple parameters, the classifiers were combined by averaging posterior probabilities [32]. The following multi-parametric classifiers were trained:

- *GM combination*: *VBM-GM* and *CBF*
- *WM combination*: *VBM-WM* and *FA*
- *Full combination*: *VBM-Brain*, *CBF*, and *FA*

For multi-class classification (AD-FTD-CN), pairwise classifiers were combined by multiplying the posterior probabilities. Using fourfold cross-validation, the mean area under the receiver operating characteristic curve (AUC), the mean accuracy, and standard deviations over 50 iterations were computed. The multi-class AUC was evaluated over pairs of classes [33], and the multi-class accuracy equalled the correctly classified rate.

Differences in mean AUC and accuracy were tested: 1) *CBF* versus *VBM-GM*, 2) *FA* versus *VBM-WM*, 3) *GM combination* versus *VBM-GM*, 4) *WM combination* versus *VBM-WM*, 5) *Full combination* versus *VBM-Brain*. This was done using non-parametric permutation tests: the difference in performance of the two classifications was compared (α ≤ 0.05) to a null distribution that was estimated using 500 permutations in which the labels were randomly distributed over the samples.

For detection of features that contributed significantly to the SVM, we calculated statistical significance maps (*p*-maps). These maps were computed on all data using an analytical expression that approximates permutation testing [34]. Clusters of significant voxels were obtained by applying a slightly conservative *p* value threshold (α ≤ 0.01). We did not correct for multiple comparisons, as permutation testing has a low false-positive detection rate [35]. The clusters’ locations were identified by visual inspection.

## Results

### Participants

**Fig. 1:**
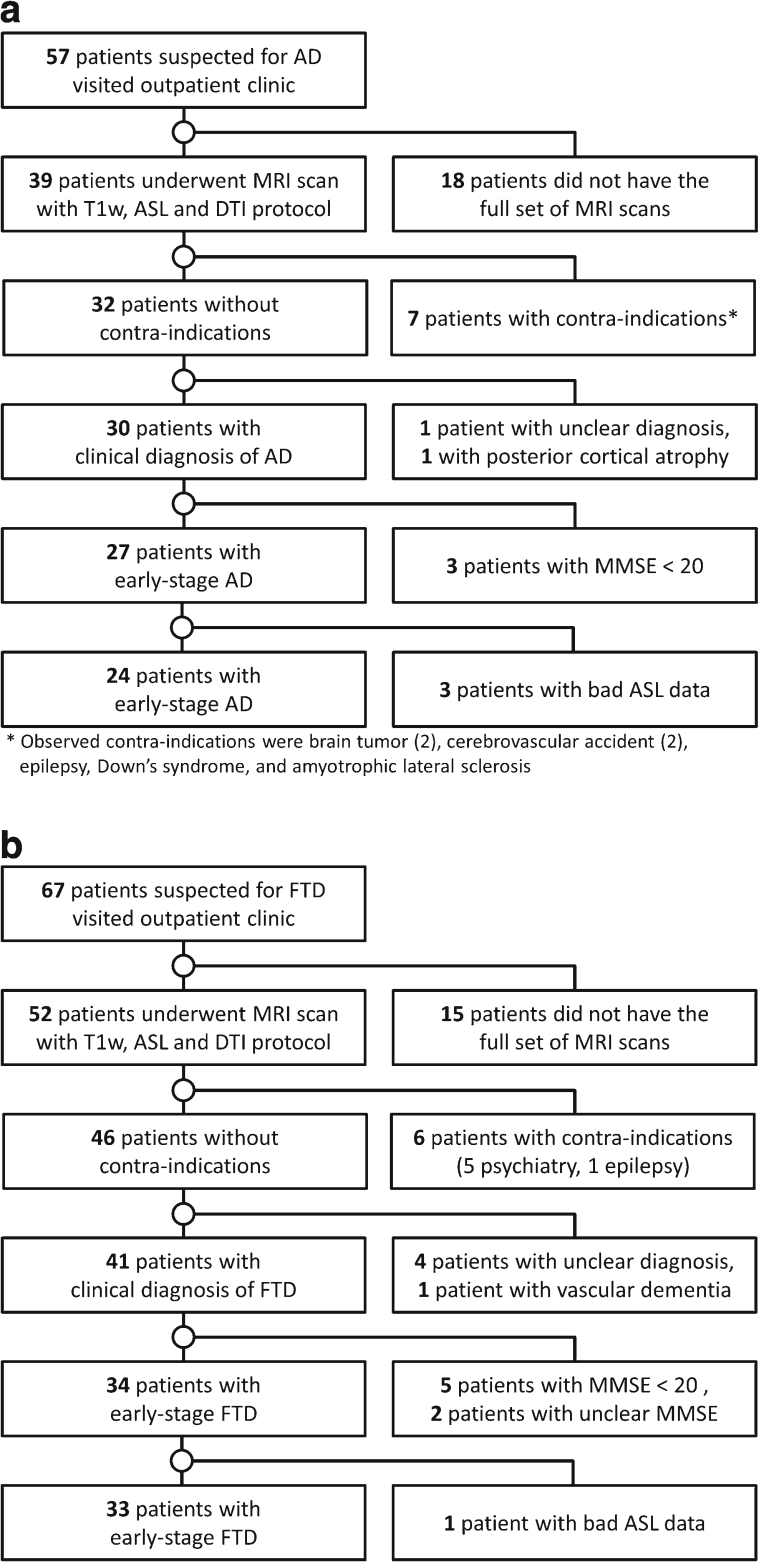
Flow of participants: **a**) patients with Alzheimer’s disease (AD), **b**) patients with frontotemporal dementia (FTD)

The inclusion of participants is visualised in Fig. 1. Table 2 shows the demographics and MMSE scores of the participants (24 AD, 33 FTD, 34 CN). Four patients were excluded because of poor ASL data quality, i.e. motion artefacts or noise. Included FTD disease subtypes were as follows: behavioural variant FTD (bvFTD, *n* = 12), PPA (*n* = 16, including ten with semantic dementia [SD] and four with progressive non-fluent aphasia [PNFA]), and five patients with unknown subtype. In the AD group, six patients had <1 year follow-up (range 0–7 months), and the diagnosis of 18 patients was confirmed by >1 year follow-up (range 12–45 months). In the FTD group, 12 patients had <1 year follow-up (range 0–11 months), and 21 patients had >1 year follow-up (range 12–47 months).

**Table 2:** Participant demographics

All participants	AD	FTD^a^	CN
No.	24	33	34
Age mean ± SD (range) [years]	67.1 ± 7.5 (52.4–81.3)	64.7 ± 8.8 (40.7–79.7)	64.7 ± 6.5 (46.5–78.8)
MMSE mean ± SD (range)^b^	24.1 ± 3.8 (15–30)^a^	25.3 ± 3.7 (15–30)^a^	28.7 ± 1.3 (25–30)
Men	AD	FTD^a^	CN
No.	15	17	22
Age mean ± SD (range) [years]	67.3 ± 7.8 (52.4–81.3)	64.5 ± 8.2 (43.5–79.7)	66.6 ± 4.3 (58.1–78.8)
MMSE mean ± SD (range)^b^	24.1 ± 4.3 (15–29)^a^	25.1 ± 4.1 (15–30)^a^	28.4 ± 1.3 (25–30)
Women	AD	FTD	CN
No.	9	16	12
Age mean ± SD (range) [years]	66.9 ± 7.4 (60.8–79.4)	64.9 ± 9.6 (40.7–78.6)	61.4 ± 8.6 (46.5–75.5)
MMSE mean ± SD (range)^b^	24.2 ± 3.2 (20–30)	25.5 ± 3.4 (20–30)	29.3 ± 1.1 (27–30)

^a^Two patients had MMSE scores of 15, which was due to language deficits. Their data were retained in the analysis, as their full neuropsychological examination indicated only moderate impairment in all cognitive domains except language

^b^The maximum score for the Mini-Mental State Examination (MMSE) is 30

*AD* Alzheimer’s disease, *CN* cognitively normal controls, *FTD* frontotemporal dementia, *MMSE* Mini-mental state examination score

### Classification results

**Fig. 2:**
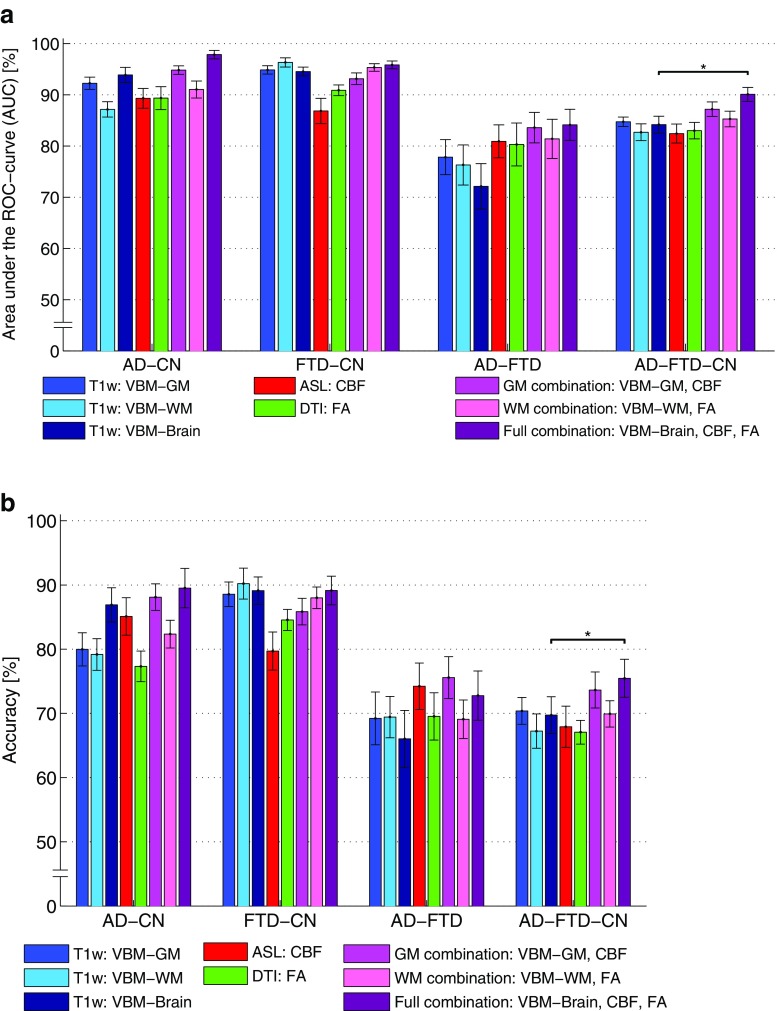
Area under the ROC curve (AUC) (**a**) and accuracy (**b**). The *error bars* show the standard deviation of 50 iterations of fourfold cross-validation. An *asterisk* (*) indicates a significant improvement over the classification using VBM features only (permutation test, *p* ≤ 0.05)

Figure 2 shows the classification performance using T1w, ASL, and DTI voxel-wise features (Fig. 2a: AUC; 2b: accuracy). Table 3 shows non-parametric testing for significant differences between classifications.

**Table 3:** *P* values of the non-parametric permutation tests to test statistical differences between classifiers based on a) mean area under the ROC curve (AUC) and b) mean accuracy

	CBF vs. VBM-GM	FA vs. VBM-WM	GM combination vs. VBM-GM	WM combination vs. VBM-WM	Full combination vs. VBM-Brain
a) Mean area under the ROC curve (AUC)					
AD-CN	0.810	0.834	0.798	0.452	0.552
FTD-CN	0.388	0.466	0.818	0.816	0.818
AD-FTD	0.752	0.668	0.472	0.322	0.052^*^
AD-FTD-CN	0.664	0.892	0.546	0.220	0.028^*^
b) Mean accuracy					
AD-CN	0.476	0.688	0.118	0.222	0.540
FTD-CN	0.210	0.324	0.624	0.462	0.998
AD-FTD	0.476	0.980	0.224	0.898	0.122
AD-FTD-CN	0.566	0.920	0.340	0.176	0.050^*^

^*^ Significant difference (*p* ≤ 0.05)

*AD* Alzheimer’s disease, *AUC* area under the receiver-operating characteristic curve, *CBF* cerebral blood flow, *CN* cognitively normal controls, *FA* fractional anisotropy, *FTD* frontotemporal dementia, *GM* grey matter, *ROC* receiver-operating characteristic, *VBM* voxel-based morphometry, *WM* white matter

For AD-CN classification, mean AUCs were 92% (*VBM-GM*), 87% (*VBM-WM*), 94% (*VBM-Brain*), 89% (*CBF*), 89% (*FA*), 95% (*GM combination*), 91% (*WM combination*), and 98% (*Full combination*). Classification accuracy was slightly lower than AUC in general. The performance using CBF and FA features was similar to that of the VBM features. The feature combinations yielded slightly higher performance than the VBM features, but differences were not significant.

For FTD-CN classification, AUCs using VBM were somewhat higher than for AD-CN, but combination with FA and CBF did not improve performance. AUCs were 95% (*VBM-GM*), 96% (*VBM-WM*), 95% (*VBM-Brain*), 87% (*CBF*), 91% (*FA*), 93% (*GM combination*), 95% (*WM combination*), and 96% (*Full combination*).

For differential diagnosis of AD versus FTD, AUCs were 78% (*VBM-GM*), 76% (*VBM-WM*), 72% (*VBM-Brain*), 81% (*CBF*), 80% (*FA*), 84% (*GM combination*), 81% (*WM combination*), and 84% (*Full combination*). Combination with CBF and FA features improved performance over the use of VBM features only.For multi-class diagnosis of AD, FTD, and CN, mean AUCs were 85% (*VBM-GM*), 83% (*VBM-WM*), 84% (*VBM-Brain*), 82% (*CBF*), 83% (*FA*), 87% (*GM combination*), 85% (*WM combination*), and 90% (*Full combination*). Classification accuracy was lower, but it should be noted that for this three-class diagnosis, the accuracy for random guessing would be only ~33%. For multi-class classification, AUCs were highest for the combination methods. The method that combined *VBM-Brain* with *CBF* and *FA* yielded a significantly higher AUC (90 vs. 84%, *p* = 0.03) and accuracy (75 vs. 70%, *p* = 0.05) than *VBM-Brain* by itself. This is reflected in the examples of confusion matrices for one iteration of the cross-validation (Appendix C; Table C1), which show a higher number of correctly classified patients and controls for *Full combination* than for *VBM-Brain*. However, combining VBM with ASL or DTI may also reduce the number of correctly classified patients, e.g. *GM Combination* has a lower number of correctly classified FTD patients than *VBM-GM*, while accuracy is improved.

### Significance maps

**Fig. 3:**
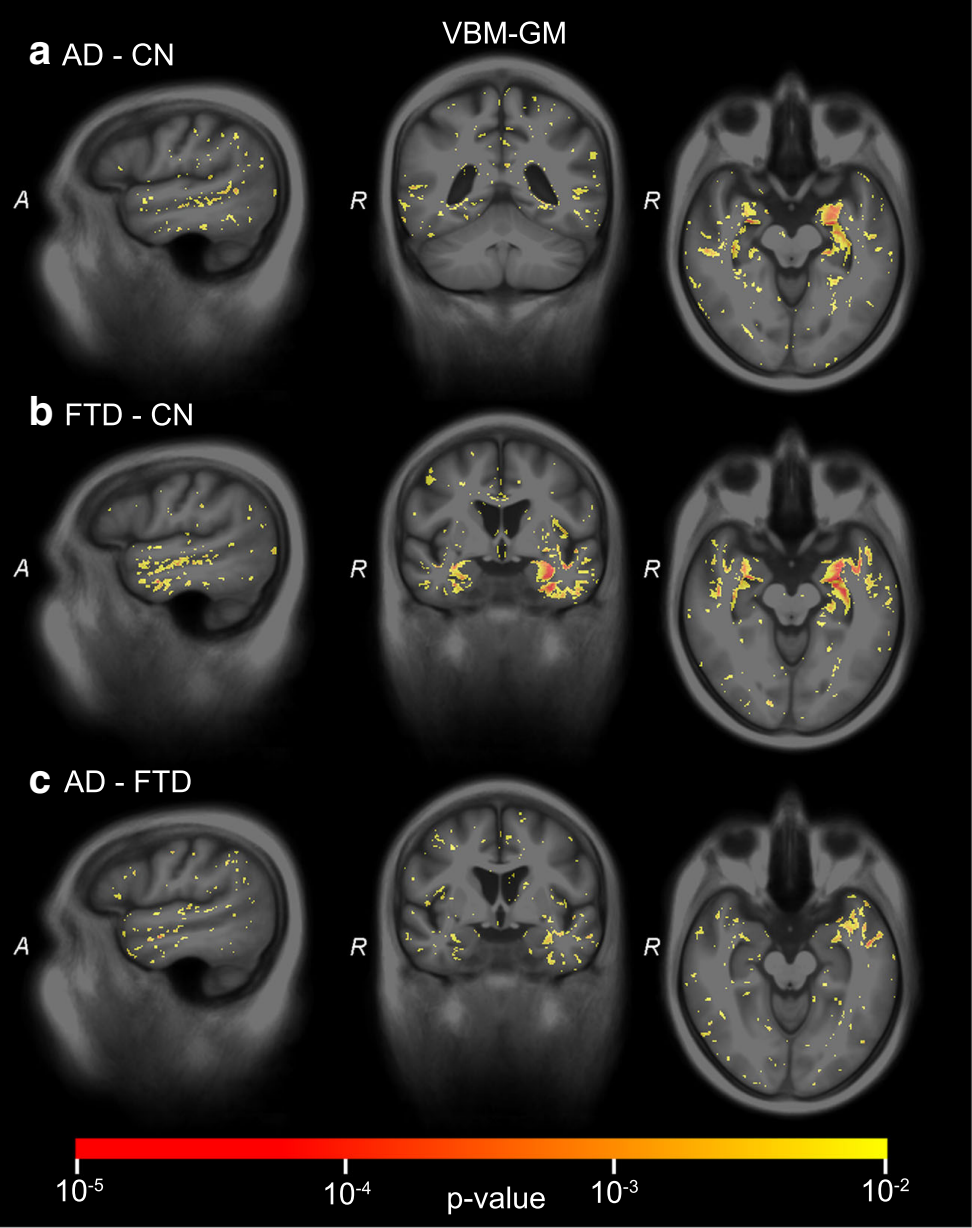
SVM significance maps for voxel-based morphometry of the grey matter (*VBM-GM*): **a**) AD-CN, **b**) FTD-CN, **c**) AD-FTD. Colour overlay shows *p* values ≤ 0.01

**Fig. 4:**
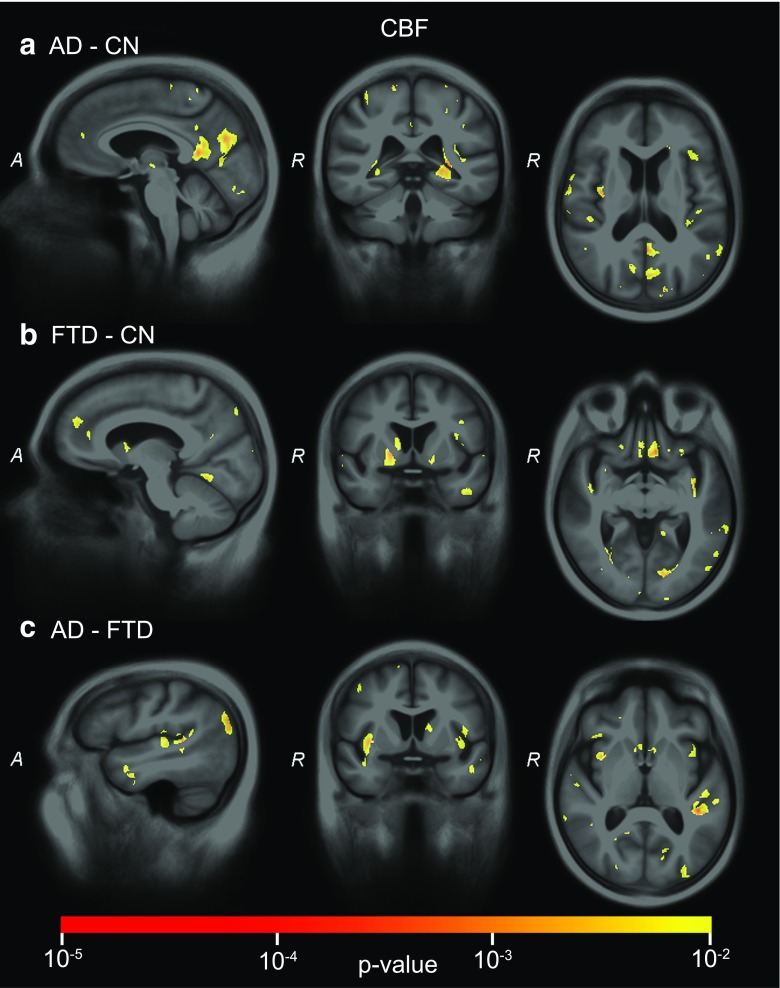
SVM significance maps for cerebral blood flow (*CBF*): **a**) AD-CN, **b**) FTD-CN, **c**) AD-FTD. Colour overlay shows *p* values ≤ 0.01

**Fig. 5:**
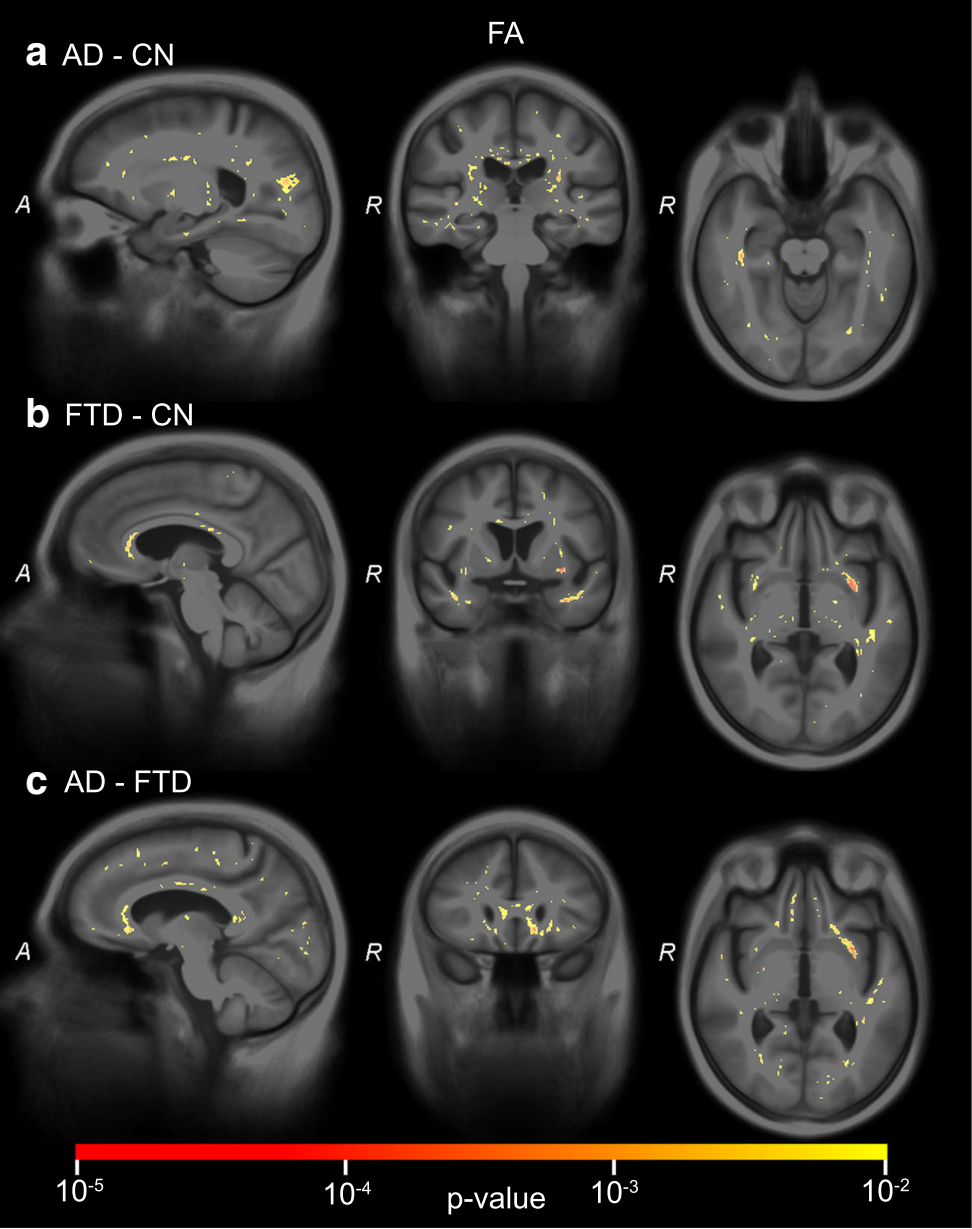
SVM significance maps for fractional anisotropy (*FA*): **a**) AD-CN, **b**) FTD-CN, **c**) AD-FTD. Colour overlay shows *p* values ≤ 0.01

Using SVM *p*-maps (Figs. 3, 4, and 5, Appendix B Figs. B1 and B2), we evaluated which voxels contributed significantly to the classifications. For *VBM-GM* (Fig. 3), we noted major influence of the perihippocampal region on the classifier; overall we observed a larger number of significant voxels in the left than in the right hemisphere. For differential diagnosis of AD-FTD, mainly voxels in the anterior temporal lobe were involved.

For *VBM-WM* (Fig. B1), we observed most clusters of significantly contributing voxels in the temporal lobe and around the ventricles. For AD-CN and FTD-CN classification, a smaller cluster of significant voxels in the corpus callosum was found. The temporal lobe clusters were present mainly in the left hemisphere, especially for AD-FTD differentiation.

For *VBM-Brain* (Fig. B2), p-maps were very smooth as the feature is formed by the Jacobian determinant of the spatially smooth deformation to template space. Smoothness is lost in *VBM-GM* and *VBM-WM* by multiplying the Jacobian determinant with the probabilistic tissue segmentations. For AD-CN, the classification was driven mainly by periventricular and left temporal lobe features. For FTD-CN, the temporal lobe contributed with the largest clusters of significant voxels. For AD-FTD, small clusters were found in the middle frontal gyrus, temporal lobe and periventricular regions.

For *CBF* (Fig. 4), p-maps showed small clusters of significant voxels in multiple brain regions. For AD-CN, significant voxels were observed mainly in the GM of the parietal lobe, precuneus, posterior cingulate gyrus, posterior temporal lobe and the insula. For FTD-CN, the main regions with significant voxels were the posterior cingulate gyrus, superior frontal gyrus, the straight gyrus, lingual gyrus and the putamen. For AD-FTD, the classification relied mainly on voxels from the posterior cingulate gyrus, parietal lobe, caudate nucleus, insula, temporal lobe and the cuneus.

For *FA* (Fig. 5), clusters of voxels in the corpus callosum and around the globus pallidus and putamen contributed significantly to the AD-CN classification. In addition, clusters of voxels in the visual and motor tracts contributed. For FTD-CN, the clusters of significant voxels were observed mainly in the anterior temporal lobe, the frontal WM, the corpus callosum, and language-associated tracts (uncinate fasciculus, superior longitudinal fasciculus). For the differential diagnosis of AD-FTD, fewer voxels were significant with only a cluster of significant voxels in the uncinate fasciculus.

## Discussion

Differential diagnosis of early-onset AD and FTD was improved (p = 0.03-0.05) by combining voxel-based features of ASL and DTI with those of structural MRI, however improvement was only borderline significant. For all classifications, ASL and DTI by themselves yielded performance similar to or slightly higher than structural MRI. While combining ASL and DTI with structural MRI improved differential diagnosis, no added value was observed for the classification of AD versus controls nor for the classification of FTD versus controls.

Classification performance was similar to that previously published on other data sets for pairwise differentiation of AD and FTD [8, 9], and slightly higher than that for multi-class classification [9]. The combination of ASL and DTI for classification of AD, FTD, and controls has not been assessed before, and therefore cannot be directly compared to literature results. The techniques have been applied separately to pairwise classifications. In concordance with our results, most studies using DTI obtained good classification performance [23, 24, 26, 27], but indicated no significant improvement over structural MRI [22, 25, 28]. In contrast to our current and previous work [19], most ASL-based classification studies showed a significant added value to structural MRI [13, 17, 18]. This is partly due to the higher performance of structural MRI in our studies. Additionally, not all studies avoid overestimation of classification performance by using cross-validation. For ASL, this overestimation might be larger than for structural MRI, because of lower signal-to-noise ratio and robustness. Conclusions obtained with or without cross-validation can therefore be expected to differ.

This work is, to the best of our knowledge, the first to perform multiparametric classification of structural MRI, ASL, and DTI. Multiparametric classification on other modalities has previously used feature-level combination (e.g. one large feature vector) or classifier-level combination (e.g. combining classifier posterior probabilities). In this study, we averaged posterior probabilities of the individual classifiers, since we had previously found this to outperform feature-level approaches [19].

The SVM significance maps showed that the brain regions contributing to the classifications corresponded to those associated with AD or FTD, which indicates that the classifier makes plausible decisions. For structural MRI, the temporal lobes showed large clusters of significant voxels. While the medial temporal lobe (i.e. hippocampus, amygdala) largely contributed to the classifications of AD versus controls and FTD versus controls, the differentiation between AD and FTD was based mainly on anterior temporal lobe features, which corresponds to the literature on atrophy in AD [27, 36–39] and FTD [27, 39]. ASL and DTI showed less influence of the temporal lobe. In the frontal and language-associated regions, DTI contributed to the classifications involving FTD. While frontal atrophy is expected in FTD [27, 39], no frontal lobe contribution was observed. ASL *p*-maps showed significant areas in the parietal lobe for classifications involving AD [40]. While parietal lobe atrophy is often proposed as a differential marker [10, 27, 39], we did not find significant clusters in the VBM *p*-maps, which is in agreement with many VBM studies, e.g. [10, 41]. In addition to the parietal lobe, CBF in the cingulate gyri and subcortical structures—insula and caudate nucleus [40]—showed significant features for AD and FTD classification. Finally, DTI captured the contribution of the corpus callosum for all classifications [20, 21]. Since the clusters of voxels influencing the classifications showed different brain regions for ASL and DTI compared to structural MRI, neuropathological processes with a spatial distribution other than atrophy are likely to be depicted.

Both the improved performances for differential diagnosis and the involvement of different brain regions suggest that ASL and DTI have additional diagnostic value to structural MRI and could improve diagnosis of individual AD and FTD patients. However, suboptimal image quality of these techniques in general, e.g. low signal-to-noise ratio, may have limited their diagnostic power when used separately. Similar to our findings, studies using data from the Alzheimer's Disease Neuroimaging Initiative 2 (ADNI 2) have shown that ASL and DTI separately provide information that is not available on structural MRI, but do not show better diagnostic power [42].

A limitation of this study is that the diagnosis was based on clinical criteria rather than post mortem histopathological examination. Although diagnosis was typically confirmed by follow-up, it is possible that some of the patients were misdiagnosed. Additionally, the size of our data set (24 AD, 33 FTD, 34 controls) was modest albeit comparable to that of other studies. Studies performing classification of AD and FTD using structural MRI data are typically of similar size [9, 13] (only larger in [8]). To obtain these group sizes, we did not limit inclusion to young-onset dementia, but included five AD and six FTD patients who were older than 70 years. In young-onset dementia, computer-aided differential diagnosis of FTD and AD would be most clinically relevant, as these patients show larger overlap of symptoms [39]. Also, we pooled the patients of several FTD subgroups (bvFTD, SD, and PNFA), which could have influenced the classification results and the regions involved in classification. The modest data size did not allow for validation on a separate validation set; instead, cross-validation was used. In addition, potential vascular white matter damage in the AD group, e.g. infarcts and white matter hyperintensities, might have influenced the classification performance of DTI. However, we expect this effect to be small, as patients were excluded when they had a history of cerebrovascular accidents (CVA) or CVA reported in their MRI examination; additionally, they were relatively young.

Regarding these limitations and the results being only borderline significant, this study primarily has exploratory value. Future research on a larger and more specific presenile cohort is needed. To assess the generalisability of our conclusions, evaluation on multi-centre data and a separate validation set is necessary as well. With our current work, we presented a computer-aided diagnosis methodology based on structural MRI, ASL, and DTI which is ready to be evaluated on a larger data set when available.

In conclusion, we postulate that ASL and DTI are promising for multiparametric computer-aided diagnosis, since combining these techniques with structural MRI improved differentiation of early-onset AD and FTD in our study.

## Electronic supplementary material

Below is the link to the electronic supplementary material.

ESM 1 (DOC 34.5 kb)

ESM 2 (DOC 23 kb)

ESM 3 (DOC 60 kb)

ESM 4 (GIF 874 kb)

High resolution image (EPS 8115 kb)

ESM 5 (GIF 773 kb)

High resolution image (EPS 7854 kb)

## Abbreviations

AD: Alzheimer’s disease
ASL: Arterial spin labelling
AUC: Area under the ROC curve
CBF: Cerebral blood flow
CN: Cognitively normal controls
DTI: Diffusion tensor imaging
FA: Fractional anisotropy
FTD: Frontotemporal dementia
GM: Grey matter
MMSE: Mini-Mental State Examination
ROC: Receiver-operating characteristic
SVM: Support vector machine
T1w: T1-weighted structural MRI
WM: White matter
VBM: Voxel-based morphometry

## References

[1] Alzheimer’s Association (2015). 2015 Alzheimer’s disease facts and figures. Alzheimers Dement.

[2] McKhann GM, Knopman DS, Chertkow H (2011). The diagnosis of dementia due to Alzheimer’s disease: recommendations from the National Institute on Aging-Alzheimer’s Association workgroups on diagnostic guidelines for Alzheimer’s disease. Alzheimers Dement.

[3] Rascovsky K, Hodges JR, Knopman D (2011). Sensitivity of revised diagnostic criteria for the behavioural variant of frontotemporal dementia. Brain.

[4] Harris JM, Thompson JC, Gall C (2015). Do NIA-AA criteria distinguish Alzheimer’s disease from frontotemporal dementia?. Alzheimers Dement.

[5] Paquerault S (2012). Battle against Alzheimer’s disease: the scope and potential value of magnetic resonance imaging biomarkers. Acad Radiol.

[6] Prince M, Bryce R, Ferri C (2011) World Alzheimer Report 2011, The benefits of early diagnosis and intervention. Alzheimer’s Disease International

[7] Klöppel S, Abdulkadir A, Jack CR (2012). Diagnostic neuroimaging across diseases. Neuroimage.

[8] Möller C, Pijnenburg YAL, Tijms B, Hafkemeijer A (2016) Alzheimer disease and behavioral variant frontotemporal dementia: automatic classification based on cortical atrophy for single-subject diagnosis. Radiology 1–11 10.1148/radiol.2015150220 26653846

[9] Raamana PR, Rosen H, Miller B (2014). Three-class differential diagnosis among Alzheimer disease, frontotemporal dementia, and controls. Front Neurol.

[10] Du A, Schuff N, Kramer J (2007). Different regional patterns of cortical thinning in Alzheimer’s disease and frontotemporal dementia. Brain.

[11] Detre JA, Leigh JS, Williams DS, Koretsky AP (1992). Perfusion imaging. Magn Reson Med.

[12] Alsop DC, Detre JA, Golay X (2015). Recommended implementation of arterial spin-labeled perfusion MRI for clinical applications: a consensus of the ISMRM perfusion study group and the European consortium for ASL in dementia. Magn Reson Med.

[13] Du A, Jahng G, Hayasaka S, Kramer J (2006). Hypoperfusion in frontotemporal dementia and Alzheimer disease by arterial spin labeling MRI. Neurology.

[14] Zhang Y, Schuff N, Ching C (2011). Joint assessment of structural, perfusion, and diffusion MRI in Alzheimer’s disease and frontotemporal dementia. Int J Alzheimer Dis.

[15] Binnewijzend MA, Kuijer JPA, van der Flier WM (2014). Distinct perfusion patterns in Alzheimer’s disease, frontotemporal dementia and dementia with Lewy bodies. Eur Radiol.

[16] Steketee RME, Bron EE, Meijboom R, Houston GC, Klein S, Mutsaerts HJMM (2016). Early-stage differentiation between presenile Alzheimer’s disease and frontotemporal dementia using arterial spin labeling MRI. Eur Radiol.

[17] Dashjamts T, Yoshiura T, Hiwatashi A (2011). Simultaneous arterial spin labeling cerebral blood flow and morphological assessments for detection of Alzheimer’s Disease. Acad Radiol.

[18] Mak HK-F, Qian W, Ng KS (2014). Combination of MRI hippocampal volumetry and arterial spin labeling MR perfusion at 3-Tesla improves the efficacy in discriminating Alzheimer’s disease from cognitively normal elderly adults. J Alzheimer Dis.

[19] Bron EE, Steketee RME, Houston GC, Oliver RA, Achterberg HC, Loog M (2014). Diagnostic classification of arterial spin labeling and structural MRI in presenile early stage dementia. Hum Brain Mapp.

[20] Zhang Y, Schuff N, Du A-T (2009). White matter damage in frontotemporal dementia and Alzheimer’s disease measured by diffusion MRI. Brain.

[21] Lu PH, Lee GJ, Shapira J (2014). Regional differences in white matter breakdown between frontotemporal dementia and early-onset Alzheimer’s disease. J Alzheimer Dis.

[22] Friese U, Meindl T, Herpertz SC (2010). Diagnostic utility of novel MRI-based biomarkers for Alzheimer’s disease: diffusion tensor imaging and deformation-based morphometry. J Alzheimer Dis.

[23] Haller S, Missonnier P, Herrmann FR (2013). Individual classification of mild cognitive impairment subtypes by support vector machine analysis of white matter DTI. Am J Neuroradiol.

[24] Besga A, Termenon M, Graña M (2012). Discovering Alzheimer’s disease and bipolar disorder white matter effects building computer aided diagnostic systems on brain diffusion tensor imaging features. Neurosci Lett.

[25] Cui Y, Wen W, Lipnicki DM (2012). Automated detection of amnestic mild cognitive impairment in community-dwelling elderly adults: a combined spatial atrophy and white matter alteration approach. Neuroimage.

[26] O’Dwyer L, Lamberton F, Bokde ALW (2012). Using support vector machines with multiple indices of diffusion for automated classification of mild cognitive impairment. PLoS One.

[27] McMillan CT, Avants BB, Cook P (2014). The power of neuroimaging biomarkers for screening frontotemporal dementia. Hum Brain Mapp.

[28] Dyrba M, Grothe M, Kirste T, Teipel SJ (2015). Multimodal analysis of functional and structural disconnection in Alzheimer’s disease using multiple kernel SVM. Hum Brain Mapp.

[29] Gorno-Tempini ML, Hillis AE, Weintraub S (2011). Classification of primary progressive aphasia and its variants. Neurology.

[30] Jones DK, Leemans A (2011). Diffusion tensor imaging. Meth Molec Biol.

[31] Chang C-C, Lin C-J (2011). LIBSVM: A library for support vector machines. ACM TIST.

[32] Tax DMJ, Van Breukelen M, Duin RPW, Kittler J (2000). Combining multiple classifiers by averaging or by multiplying?. Pattern Recognit.

[33] Hand DJ, Till RJ (2001). A simple generalisation of the area under the ROC curve for multiple class classification problems. Mach Learn.

[34] Gaonkar B, Shinohara RT, Davatzikos C (2015). Interpreting support vector machine models for multivariate group wise analysis in neuroimaging. Med Image Anal.

[35] Gaonkar B, Davatzikos C (2013). Analytic estimation of statistical significance maps for support vector machine based multi-variate image analysis and classification. Neuroimage.

[36] Bastos Leite A, Scheltens P, Barkhof F (2004). Pathological aging of the brain: an overview. Top Magn Reson Imaging.

[37] Frisoni G, Testa C, Zorzan A (2002). Detection of grey matter loss in mild Alzheimer’s disease with voxel based morphometry. J Neurol Neurosurg Psychiatry.

[38] Chételat G, Desgranges B, De La Sayette V (2002). Mapping gray matter loss with voxel-based morphometry in mild cognitive impairment. Neuroreport.

[39] Seelaar H, Rohrer JD, Pijnenburg YAL (2011). Clinical, genetic and pathological heterogeneity of frontotemporal dementia: a review. J Neurol Neurosurg Psychiatry.

[40] Hu WT, Wang Z, Lee V-Y (2010). Distinct cerebral perfusion patterns in FTLD and AD. Neurology.

[41] Whitwell JL, Josephs KA, Rossor MN (2005). Magnetic resonance imaging signatures of tissue pathology in frontotemporal dementia. Arch Neurol.

[42] Jack CR, Barnes J, Bernstein MA (2015). Magnetic resonance imaging in Alzheimer’s Disease Neuroimaging Initiative 2. Alzheimers Dement.

