# Supplementary material for: Multiparametric computer-aided differential diagnosis of Alzheimer’s disease and frontotemporal dementia using structural and advanced MRI
Source: Eur Radiol. 2016 Dec 16;27(8):3372–82. doi: 10.1007/s00330-016-4691-x (PMC5491625; doi:10.1007/s00330-016-4691-x)
Supplement: Supplementary file 1 — (DOC 34.5 kb) [file 330_2016_4691_MOESM1_ESM.doc]

Appendix A

The image processing pipeline of [1], the *Iris pipeline*, was extended and applied. All registrations were performed using Elastix registration software [2, 3] by maximizing mutual information [4]. The following sections detail the image processing steps of the *Iris pipeline* [1] to obtain voxel-based features of structural MRI, arterial spin labelling (ASL), and diffusion tensor imaging (DTI).

Structural MRI

Probabilistic tissue segmentations were obtained for white matter (WM), grey matter (GM), and cerebrospinal fluid (CSF) on the structural T1-weighted (T1w) image using SPM8 (Statistical Parametric Mapping, London, UK) [5].

Individual brain masks were constructed using multi-atlas segmentation. As a first step, we applied the Brain Extraction Tool (BET) [6] to the T1w images associated with a set of 30 atlases [7, 8]. We checked the BET brain masks visually and adjusted the extraction parameters if needed. Second, the 30 atlas images were registered to each subject's non-uniformity-corrected T1w image [9]. These registrations were initialised using rigid registrations of the BET masks of the subject’s image and the atlas images, and used a rigid, affine, and a non-rigid B-spline transformation model consecutively. Third, the BET brain masks were transformed using the obtained transformation parameters and were fused with majority voting [10], resulting in a brain mask for each subject. These brain masks were used for ASL partial volume correction and intracranial volume estimation.

A group template space was constructed based on the T1w images of all subjects using a procedure that avoids bias towards any of the individual T1w images [1]. In this approach, the coordinate transformations from the template space to the subject's T1w space were derived from pairwise image registrations of all pairs of T1w images. For these pairwise image registrations, we used T1w images that were non-uniformity-corrected and skull-stripped using the multi-atlas brain mask explained above. The pairwise registrations were performed using a similarity, affine, and non-rigid B-spline transformation model consecutively. A similarity transformation is a rigid transformation including isotropic scaling. The non-rigid B-spline registration used a three-level multiresolution framework with isotropic control-point spacing of 24, 12, and 6 mm at the three resolution levels, respectively. For the extraction of the features, all images, masks, and segmentations were transformed to this group template space.

In the group template space, we derived T1w features based on voxel-based morphometry (VBM) using 1) the probabilistic GM segmentation (*VBM-GM*), 2) the probabilistic WM segmentation (*VBM-WM*) and 3) the brain mask (*VBM-Brain*). These segmentations were modulated, i.e. multiplied by the Jacobian determinant of the deformation field, to take compression and expansion into account [11]. This modulation step ensured that the overall brain volume was not changed by the transformation to template space. For the final feature maps, the cerebellum and brain stem were masked out using majority vote of the transformed atlases.

ASL

ASL imaging data consisted of a perfusion-weighted image (*ΔM*) and a proton density normalization image (*M_0_*). The probabilistic GM segmentation was rigidly registered to the *ΔM* image to obtain the ASL-T1w transformation. WM and CSF segmentations and brain masks were transformed to ASL space accordingly. Cerebral blood flow (CBF) was quantified using the single-compartment model proposed by Buxton et al. [1998], which is the recommended approach for pseudo-continuous ASL [13]. The labelling efficiency α [14] was corrected for background suppression pulses [15], resulting in α = 0.8 x 0.75 = 0.6. Other parameters were T1_GM_=1.6 s, and blood-brain partition coefficient λ_GM_= 0.95 mL/g. CBF was quantified in GM only. For partial volume correction, a 3D method was applied based on local linear regression using the tissue probability maps [16, 17]. CBF maps were transformed to T1w template space in one pass by concatenating the template-T1w transformation and the inverted ASL-T1w transformation. The CBF voxel values in GM in the template space were used as features for classification.

DTI

Diffusion-weighted data were corrected for motion and eddy currents by affine registration of the diffusion-weighted volumes to the average of the three b_0_ volumes [18]. The rotation component of each transformation was used to realign each gradient vector. Transformed diffusion-weighted images were resampled at an isotropic resolution of 1.0 mm. A brain mask was created using BET and multi-atlas segmentation based on three DTI atlases. Tensor fits were performed with a weighted least squares optimization using the DTIfit tool of the FMRIB Software Library (FSL) [19]. Fractional anisotropy (FA) maps were computed from the tensor images.

The mean b_0_ image was registered with the T1w image using an affine transformation model. Rigid registration on the brain masks was used for initialization of this registration. FA maps were transformed to T1w template space in one pass by concatenating the template-T1w and T1w-DTI transformations. The FA voxel values in WM in the template space were used as features for classification.

References

1. Bron EE, Steketee RME, Houston GC, et al (2014) Diagnostic classification of arterial spin labeling and structural MRI in presenile early stage dementia. Hum Brain Mapp 35:4916–4931. doi: 10.1002/hbm.22522.

2. Klein S, Staring M, Murphy K, et al (2010) Elastix: a toolbox for intensity-based medical image registration. IEEE Trans Med Imaging 29:196–205.

3. Shamonin DP, Bron EE, Lelieveldt BP, et al (2014) Fast parallel image registration on CPU and GPU for diagnostic classification of Alzheimer’s disease. Front Neuroinform 7:1–15. doi: 10.3389/fninf.2013.00050

4. Thévenaz P, Unser M (2000) Optimization of mutual information for multiresolution image registration. IEEE Trans Image Proc 9:2083–2099.

5. Ashburner J, Friston KJ (2005) Unified segmentation. Neuroimage 26:839–851. doi: 10.1016/j.neuroimage.2005.02.018

6. Smith SM (2002) Fast robust automated brain extraction. Hum Brain Mapp 17:143–155.

7. Gousias IS, Rueckert D, Heckemann RA, et al (2008) Automatic segmentation of brain MRIs of 2-year-olds into 83 regions of interest. Neuroimage 40:672–684.

8. Hammers A, Allom R, Koepp MJ, et al (2003) Three-dimensional maximum probability atlas of the human brain, with particular reference to the temporal lobe. Hum Brain Mapp 19:224–247.

9. Tustison NJ, Avants BB, Cook PA, et al (2010) N4ITK: improved N3 bias correction. IEEE Trans Med Imaging 29:1310–1320.

10. Heckemann RA, Hajnal J V, Aljabar P, et al (2006) Automatic anatomical brain MRI segmentation combining label propagation and decision fusion. Neuroimage 33:115–126.

11. Ashburner J, Friston KJ (2000) Voxel-based morphometry - the methods. Neuroimage 11:805–821.

12. Buxton RB, Frank LR, Wong EC, et al (1998) A general kinetic model for quantitative perfusion imaging with arterial spin labeling. Magn Reson Med 40:383–396.

13. Alsop DC, Detre JA, Golay X, et al (2015) Recommended implementation of arterial spin-labeled perfusion MRI for clinical applications: A consensus of the ISMRM perfusion study group and the European consortium for ASL in dementia. Magn Reson Med 73:102–116. doi: 10.1002/mrm.25197

14. Aslan S, Xu F, Wang PL, et al (2010) Estimation of labeling efficiency in pseudocontinuous arterial spin labeling. Magn Reson Med 63:765–771. doi: 10.1002/mrm.22245

15. Garcia DM, Duhamel G, Alsop DC (2005) Efficiency of inversion pulses for background suppressed arterial spin labeling. Magn Reson Med 54:366–372. doi: 10.1002/mrm.20556

16. Asllani I, Borogovac A, Brown TR (2008) Regression algorithm correcting for partial volume effects in arterial spin labeling MRI. Magn Reson Med 60:1362–1371.

17. Oliver RA, Thomas DL, Golay X (2012) Improved partial volume correction of ASL images using 3D kernels. ISMRM Br. Chapter

18. De Groot M, Verhaaren BFJ, de Boer R, et al (2013) Changes in normal-appearing white matter precede development of white matter lesions. Stroke 44:1037–1042. doi: 10.1161/STROKEAHA.112.680223

19. Behrens TEJ, Woolrich MW, Jenkinson M, et al (2003) Characterization and propagation of uncertainty in diffusion-weighted MR imaging. Magn Reson Med 50:1077–1088. doi: 10.1002/mrm.10609
