# Supplementary material for: Multiparametric computer-aided differential diagnosis of Alzheimer’s disease and frontotemporal dementia using structural and advanced MRI
Source: Eur Radiol. 2016 Dec 16;27(8):3372–82. doi: 10.1007/s00330-016-4691-x (PMC5491625; doi:10.1007/s00330-016-4691-x)
Supplement: Supplementary file 2 — (DOC 23 kb) [file 330_2016_4691_MOESM2_ESM.doc]

Appendix B

**Fig. B1** SVM significance maps for voxel-based morphometry of the white matter (*VBM-WM*): A) AD-CN, B) FTD-CN, C) AD-FTD. Colour overlay shows *p* values ≤ 0.01

**Fig. B2** SVM significance maps for voxel-based morphometry of the supratentorial brain (*VBM-Brain*): A) AD-CN, B) FTD-CN, C) AD-FTD. Colour overlay shows *p* values ≤ 0.01
