# Supplementary material for: Multiparametric computer-aided differential diagnosis of Alzheimer’s disease and frontotemporal dementia using structural and advanced MRI
Source: Eur Radiol. 2016 Dec 16;27(8):3372–82. doi: 10.1007/s00330-016-4691-x (PMC5491625; doi:10.1007/s00330-016-4691-x)
Supplement: Supplementary file 3 — (DOC 60 kb) [file 330_2016_4691_MOESM3_ESM.doc]

Appendix C

**Table C1**

Confusion matrices for the multi-class classification of CN, FTD, and AD. Results show an iteration of the fourfold cross-validation

| ***VBM-GM*** | | True | | |  | ***GM Combination***  *(VBM-GM + CBF)* | | True | | |
| --- | --- | --- | --- | --- | --- | --- | --- | --- | --- | --- |
|  |  | CN | FTD | AD |  |  |  | CN | FTD | AD |
| Predicted | CN | 29 | 1 | 3 |  | Predicted | CN | 31 | 3 | 5 |
|  | FTD | 1 | 26 | 10 |  |  | FTD | 1 | 24 | 7 |
|  | AD | 4 | 6 | 11 |  |  | AD | 1 | 6 | 12 |
|  |  |  |  |  |  |  |  |  |  |  |
| ***VBM-WM*** | | True | | |  | ***WM Combination***  *(VBM-WM + FA****)*** | | True | | |
|  |  | CN | FTD | AD |  |  |  | CN | FTD | AD |
| Predicted | CN | 26 | 2 | 7 |  | Predicted | CN | 30 | 2 | 7 |
|  | FTD | 1 | 27 | 10 |  |  | FTD | 4 | 28 | 10 |
|  | AD | 7 | 4 | 7 |  |  | AD | 0 | 3 | 7 |
|  | |  | | |  |  |  |  |  |  |
| ***VBM-Brain*** | | True | | |  | ***Full Combination***  *(VBM-Brain + CBF + FA)* | | True | | |
|  |  | CN | FTD | AD |  |  |  | CN | FTD | AD |
| Predicted | CN | 30 | 2 | 4 |  | Predicted | CN | 33 | 2 | 6 |
|  | FTD | 0 | 25 | 12 |  |  | FTD | 0 | 28 | 9 |
|  | AD | 4 | 6 | 8 |  |  | AD | 1 | 3 | 9 |
|  | |  | | |  |  |  |  |  |  |
| ***CBF*** | | True | | |  |  |  |  |  |  |
|  |  | CN | FTD | AD |  |  |  |  |  |  |
| Predicted | CN | 28 | 6 | 4 |  |  |  |  |  |  |
|  | FTD | 3 | 22 | 5 |  |  |  |  |  |  |
|  | AD | 3 | 5 | 15 |  |  |  |  |  |  |
|  | |  | | |  |  |  |  |  |  |
| ***FA*** | | True | | |  |  |  |  |  |  |
|  |  | CN | FTD | AD |  |  |  |  |  |  |
| Predicted | CN | 30 | 4 | 8 |  |  |  |  |  |  |
|  | FTD | 4 | 26 | 11 |  |  |  |  |  |  |
|  | AD | 0 | 3 | 5 |  |  |  |  |  |  |
|  | |  | | |  |  |  |  |  |  |

*AD* Alzheimer’s disease, *CBF* cerebral blood flow, *CN* cognitively normal controls, *FA* fractional anisotropy, *FTD* frontotemporal dementia, *GM* grey matter, *VBM* voxel-based morphometry, *WM* white matter
