# Supplementary figures and images for: Multiparametric computer-aided differential diagnosis of Alzheimer’s disease and frontotemporal dementia using structural and advanced MRI
Source: Eur Radiol. 2016 Dec 16;27(8):3372–82. doi: 10.1007/s00330-016-4691-x (PMC5491625; doi:10.1007/s00330-016-4691-x)

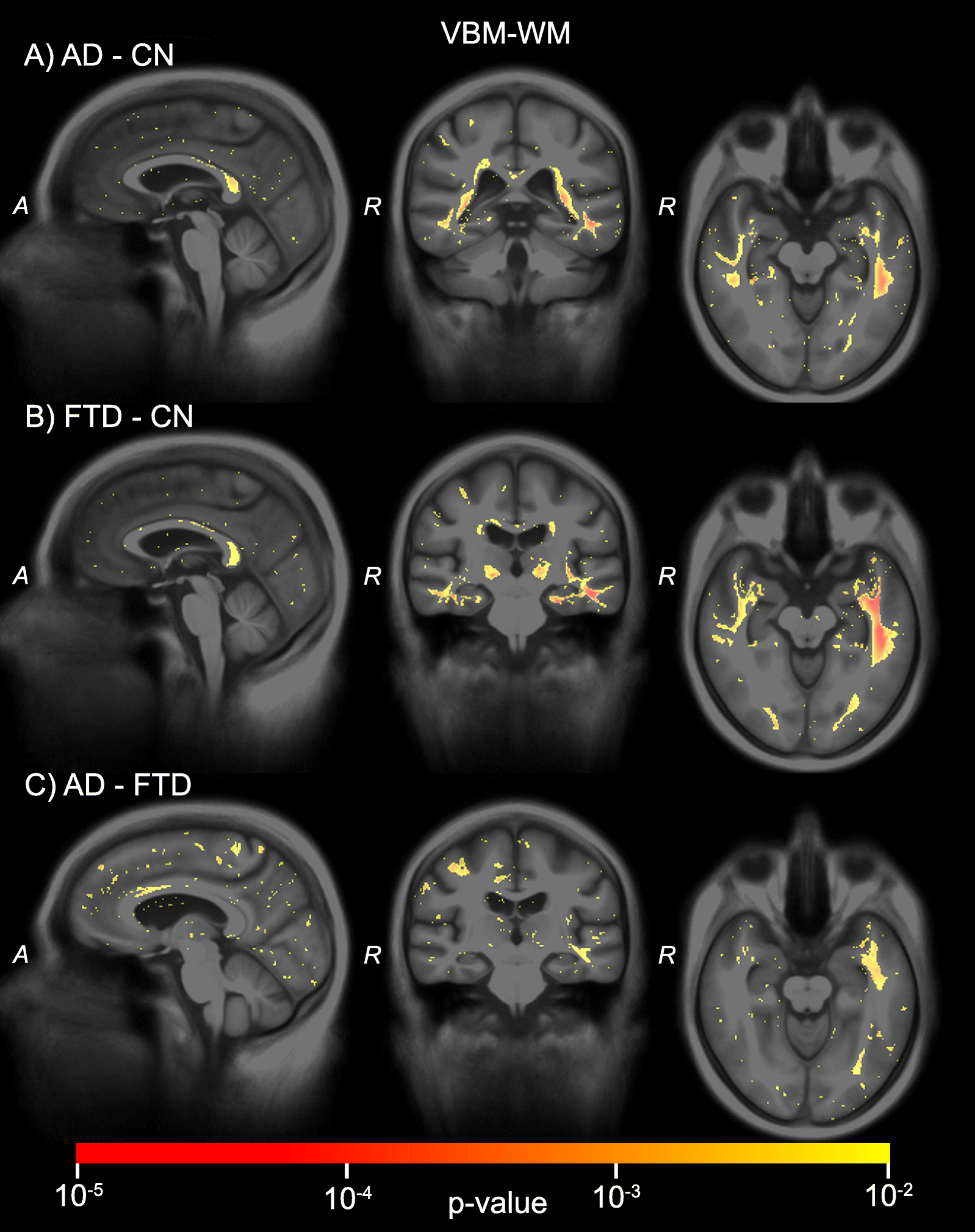

Supplement: Supplementary file 4 — (GIF 874 kb) [file 330_2016_4691_Fig6_ESM.gif]

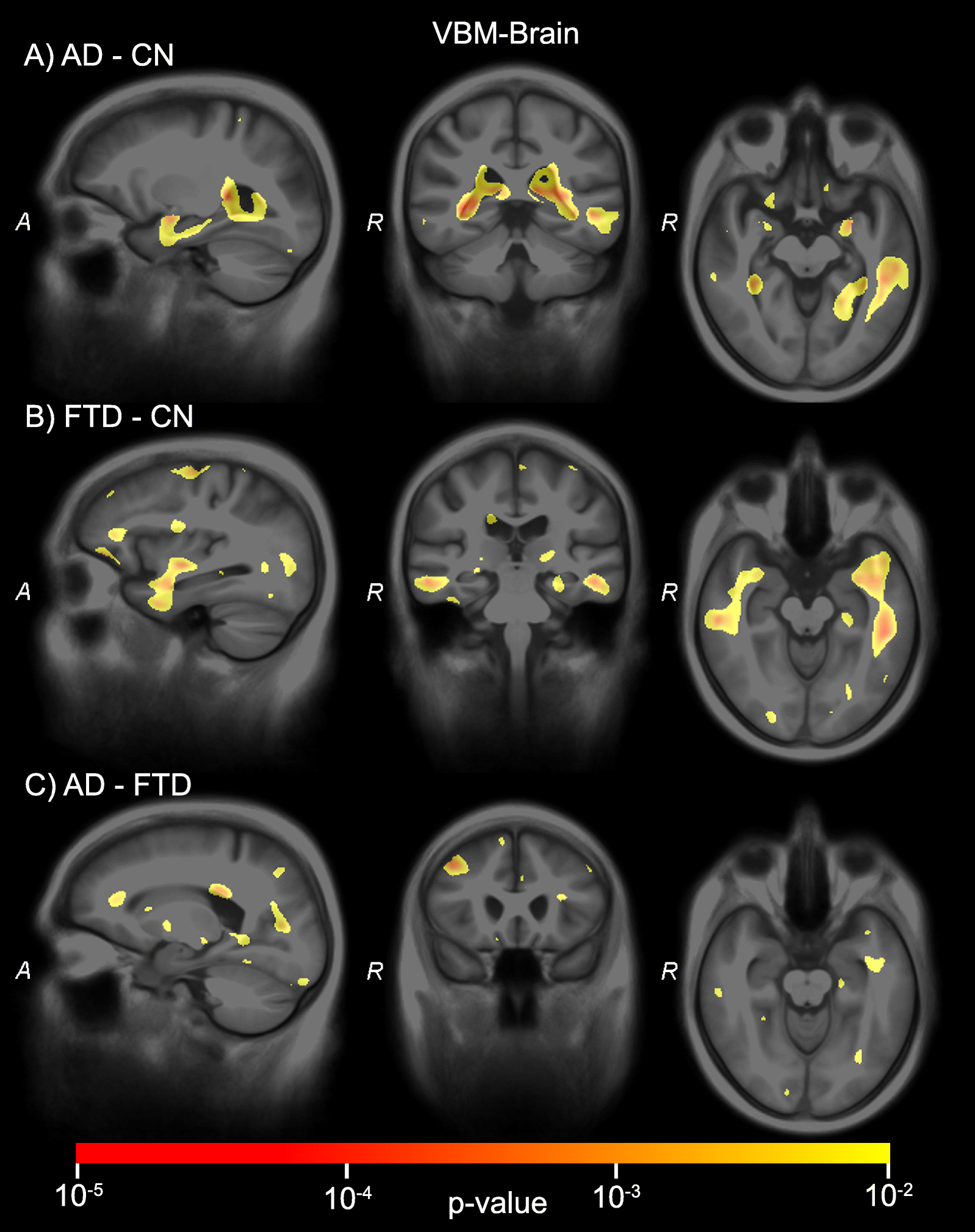

Supplement: Supplementary file 6 — (GIF 773 kb) [file 330_2016_4691_Fig7_ESM.gif]
